# Comanagement With Nephrologist Care Is Associated With Fewer Cardiovascular Events Among Liver Transplant Recipients With Chronic Kidney Disease

**DOI:** 10.1097/TXD.0000000000001220

**Published:** 2021-09-20

**Authors:** Patrick T. Campbell, Megan Kosirog, Blessing Aghaulor, Dyanna Gregory, Stewart Pine, Amna Daud, Arighno Das, Daniel J. Finn, Josh Levitsky, Jane L. Holl, Donald M. Lloyd-Jones, Lisa B. VanWagner

**Affiliations:** 1 Division of Gastroenterology and Hepatology, Department of Medicine, Northwestern University, Chicago, IL.; 2 Comprehensive Transplant Center, Northwestern University Feinberg School of Medicine, Chicago, IL.; 3 Center for Healthcare Delivery Science and Innovation, University of Chicago Medicine, Chicago, IL.; 4 Biological Sciences Division, Department of Neurology, University of Chicago, Chicago, IL.; 5 Department of Preventive Medicine, Northwestern University Feinberg School of Medicine, Chicago, IL.; 6 Division of Cardiology, Department of Medicine, Northwestern University Feinberg School of Medicine, Chicago, IL.

## Abstract

**Background.:**

Chronic kidney disease (CKD) is associated with cardiovascular (CV) events, a leading complication in liver transplant recipients (LTRs). Timely subspecialty care is associated with improved clinical outcomes in patients with CKD. This study sought to assess associations between nephrology comanagement and CV events among LTRs at risk for or with CKD.

**Methods.:**

LTRs with CKD plus those at risk were identified in an inception cohort at a single tertiary care network between 2010 and 2016, using electronic health record data and manual chart review. CKD was defined as estimated glomerular filtration rate <60 mL/min/1.73 m^2^ or International Classification of Diseases 9th or 10th revision code for CKD and at-risk CKD as estimated glomerular filtration rate 60–89 mL/min/1.73 m^2^. Cox proportional hazard models assessed the association between nephrology comanagement and CV events among LTRs with or at risk for CKD.

**Results.:**

Among 602 LTRs followed for up to 6 y posttransplant, prevalence of CKD plus those at risk increased yearly (71% in year 1, 86% in year 6) (*P* < 0.0001). Rates of nephrology comanagement decreased yearly posttransplant (35% in year 1, 28% in year 6). In multivariable models, nephrology comanagement was associated with lower CV events (adjusted hazard ratio, 0.57; 95% confidence interval, 0.33–0.99).

**Conclusions.:**

Among LTRs with CKD, nephrology comanagement may be associated with lower CV events. A prospective study is needed to identify the reasons for improved outcomes and barriers to nephrology referral.

## Abstract 2

Supplemental Digital Content is available in the text.

## Graphical abstract

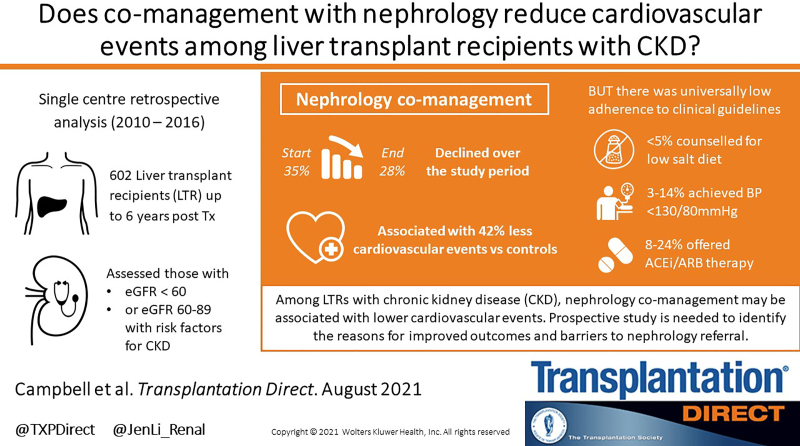

## INTRODUCTION

Chronic kidney disease (CKD) is a common complication after liver transplant (LT), with an incidence of stage 4 or 5 CKD (defined as estimated glomerular filtration rate [eGFR], <30 mL/min per 1.73 m^2^) up to 18% at 5 y after LT.^1^ Many factors have been associated with increased risk of CKD post-LT, including pretransplant renal dysfunction, diabetes, hypertension, and immunosuppression with calcineurin inhibitors.^1,2^

CKD is a well-known risk factor for cardiovascular (CV) disease in the general population.^3^ Among LT recipients (LTRs), CV disease is a leading cause of mortality, and renal dysfunction at the time of and immediately posttransplant is associated with higher risk of major CV events.^4,5^ In addition, other factors that have been associated with increased mortality after LT include pre-LT CKD, post-LT acute renal failure, and post-LT CKD.^6-8^ Given the significant CKD burden in this patient population and the known negative effects of this condition on clinical outcomes, improvement in the management of CKD could have significant potential to improve clinical outcomes for LTRs.

In the general CKD population, prior data have shown that early nephrology referral for management of CKD is associated with lower hospitalizations and mortality rates.^9^ Consequently, current clinical practice guidelines for the general CKD population recommend nephrology referral for several clinical scenarios.^10^ These guidelines also provide recommendations on management of both CKD and many other chronic conditions that frequently occur in patients with CKD including hypertension, hyperlipidemia, diabetes, anemia, etc. Although the aforementioned clinical practice guidelines help guide management of patients with CKD, published guidance on the management of CKD among LTRs has focused on adapting key aspects from the nontransplant CKD guidelines that are most relevant to the transplant population, such as adjustment of immunosuppressive regimens to limit progression of CKD and therapeutic considerations to minimize drug–drug interactions for management of highly prevalent comorbid conditions (eg, diabetes, hypertension, proteinuria) in transplant recipients.^11^

Despite the breadth of recommendations for CKD management and high prevalence of CKD in LTRs, data on transplant provider management of CKD among LTRs are limited. Therefore, this study sought to assess rates of nephrology comanagement for CKD and utilization of guideline-recommended medical therapy among LTRs with CKD and whether these metrics are associated with a reduction in CV events.

## MATERIALS AND METHODS

### Study Design

A longitudinal inception cohort study was conducted at a large, urban, tertiary care network in the United States. The institutional review board of Northwestern University approved the study.

### Study Population

Patients who underwent LT between January 1, 2010, and December 31, 2016, were included in the study. We excluded patients who died within the first 6 mo after LT in an attempt to study patients with stable immunosuppression and graft function.

### Data Source and Collection

Eligible LTRs were identified using International Classification of Diseases 9th or 10th Revision (ICD-9/10) codes and clinical information was obtained from the Northwestern Medicine Enterprise Data Warehouse, which contains comprehensive demographic, clinical, diagnostic, procedural, and administrative data for 7.5 million unique patients from all Northwestern Medicine sites. In addition, manual chart review was used for data that are not easily captured in an electronic health record (EHR), such as clinical reasoning for not adhering to a guideline recommendation (eg, documentation of why it would be inappropriate given the unique clinical scenario). Vital status was obtained from the Organ Procurement and Transplantation Network database, which is linked to the US Social Security Death Index. Data were linked to clinical data of each LTR based on a previously published methodology.^5,12^

### CKD and Covariate Definitions

Renal function was assessed by serum creatinine and corresponding eGFR calculated by the Modification of Diet in Renal Disease 4-variable equation.^13^ CKD was identified by ICD-9/10 code or by using eGFR on at least 2 separate outpatient visits separated by ≥90 d.^14,15^ CKD was defined as eGFR <60 mL/min/1.73 m^2^. CKD plus those at risk was defined as eGFR <90 mL/min/1.73 m^2^. At risk for CKD was defined as eGFR 60–89 mL/min/1.73 m^2^. Renal replacement status pre- and post-LT was defined as at least 2 encounters for intermittent hemodialysis or continuous renal replacement therapy before or after LT. Patients undergoing renal replacement were assigned an eGFR of 0 mL/min/1.73 m^2^, given the serum creatinine did not reflect actual renal function. Hypertension was identified by ICD-9/10 code, order of blood pressure (BP)-lowering medication, or systolic BP ≥140 or diastolic BP ≥90 mm Hg on at least 2 separate outpatient visits, consistent with clinical practice guidelines during the study period. Diabetes was identified by ICD-9/10 code, hemoglobin A1C ≥6.5%, random blood glucose >200 mg/dL, or use of glucose-lowering medication, in the setting of prednisone daily dose ≤10 mg. Atherosclerotic cardiovascular disease (ASCVD) was identified by ICD-9/10 code for acute coronary syndrome, myocardial infarction, stable or unstable angina, coronary or other arterial revascularization, stroke, or transient ischemic attack. Obesity was defined as body mass index ≥30 kg/m^2^ or ICD-9/10 code. Hyperlipidemia was identified by ICD-9/10 code, treatment with lipid-lowering medications, or total cholesterol ≥200 mg/dL. The standard model for end-stage liver disease (MELD) score was used because this was the allocation system in use during the study period. The immunosuppression and clinical visit protocol at our institution has been described previously.^16^

### Assessment of CKD Management

We used manual chart review to assess rates of nephrology comanagement among LTRs. Nephrology comanagement of CKD was defined as any referral to a nephrology specialist placed by a transplant provider during the study period or if a patient was already under the care of a nephrologist during the study period. We also assessed guideline-recommended medication use and average annual BP, using both manual and electronic chart review as described previously.^16^

### Exposure and Outcome Measures

The primary exposure variable was comanagement of CKD plus at-risk CKD by a nephrology specialist. The primary outcome variable was a CV event, defined as death from a CV cause or hospitalization for myocardial infarction/revascularization, cardiac arrest, heart failure, atrial fibrillation, thromboembolism, or stroke. In secondary analysis, we examined differences in rates of comanagement, process measures for CVD care, and associations with CV events among the subgroups with CKD (eGFR, <60) and those at risk for CKD (eGFR, 60–89).

### Statistical Analysis

A *t* test, chi-square test, or Fisher exact test was used to examine group differences by nephrology comanagement status for continuous or categorical variables, as appropriate. Study participants without a serum creatinine recorded post-LT were excluded from this analysis (n = 30). Cox proportional hazard models were used to estimate major CV events from time of LT between LTRs with CKD who were comanaged by a nephrologist and those who were not. Time of LT was taken as time zero as the majority of CKD diagnoses occurred within the first year of LT and a substantial proportion (20%) of patients were already under the care of a nephrologist at the time of LT. The proportional hazard assumption was met and residuals were normally distributed. The model was adjusted a priori for sex, race, age at transplant and time-varying diabetes, ASCVD, use of BP-lowering medication, and CKD stage. SAS software version 9.4 (SAS Institute, Cary, NC) was used to complete all analyses. All *P* values are 2-sided and a *P* < 0.05 was considered to indicate statistical significance.

## RESULTS

### Demographics

**FIGURE 1.**
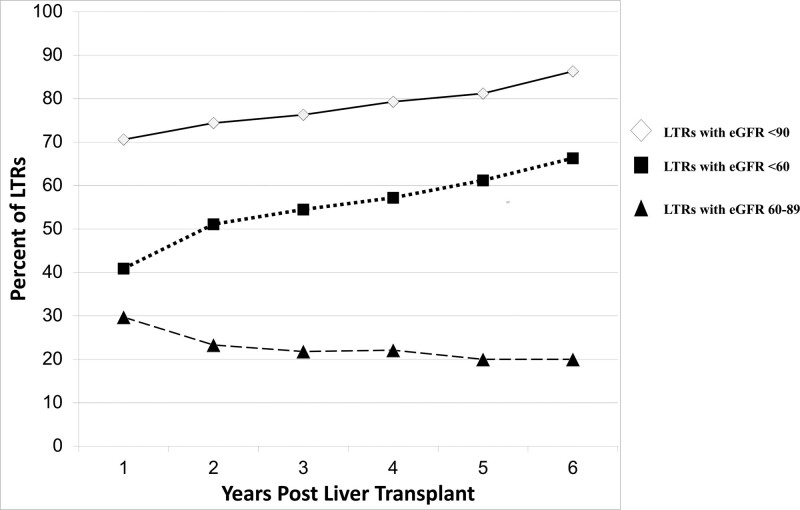
Prevalence of CKD plus those at risk (eGFR, <90 mL/min/1.73 m^2^), CKD (eGFR, <60 mL/min/1.73 m^2^), and those at risk for CKD (eGFR, 60–89 mL/min/1.73 m^2^) by year from liver transplant. CKD, chronic kidney disease; eGFR, estimated glomerular filtration rate; LTR, liver transplant recipient.

Among the 602 patients transplanted during the study period that survived at least 6 mo and had a minimum of 1-y follow-up time, 79.1% (n = 476) met criteria for diagnosis of CKD (eGFR, <60 mL/min/1.73 m^2^) or at risk for CKD (eGFR, <60–89 mL/min/1.73 m^2^) during the study time period. Out of these 476 patients, 416 met criteria for CKD (eGFR, <60 mL/min/1.73 m^2^) and 60 met criteria for at risk for CKD (eGFR, <60–89 mL/min/1.73 m^2^). The prevalence of CKD plus those at risk (eGFR, <90 mL/min/1.73 m^2^) at time of LT was 41.5% and increased yearly post-LT from 71% in year 1 to 86% in year 6 (*P* < 0.0001). The prevalence of CKD (eGFR, < 60 mL/min/1.73 m^2^) also increased yearly post-LT from 41% in year 1 to 66% in year 6 (*P* < 0.0001) (Figure 1).

Cohort characteristics of LTRs with CKD or at risk for CKD stratified by nephrology comanagement status are shown in Table 1. The average age at time of LT was 57 ± 11 y, 60.7% of LTRs were men, 64.1% of LTRs identified as non-Hispanic White, and 17.9% were of Hispanic ethnicity. The most common indication for LT was hepatitis C (32.4%), followed by alcohol (22.1%), autoimmune (13.1%), and nonalcoholic steatohepatitis (12.9%). The mean MELD score at transplant was 24. LTRs with CKD or at risk for CKD who were comanaged with nephrology had higher rates of comorbid diabetes (43% versus 30%, *P* = 0.006) and higher mean MELD at time of LT (27.1 versus 21.7, *P* < 0.0001) compared with those that were not. There was no difference in prevalence of hypertension (59% with nephrology comanagement versus 53% without, *P* = 0.16), mean systolic BP level (133.9 mm Hg with comanagement versus 131.9 mm Hg without, *P* = 0.051), or BP medication usage (59% with comanagement versus 61% without, *P* = 0.78) by nephrology comanagement status.

**TABLE 1.** Cohort characteristics of LT recipients with or at risk for CKD^*a*^ (n = 476) by nephrology comanagement status at time of liver transplant

	With or at risk for CKD(n = 476)	Nephrology comanagement(n = 202)	No nephrology comanagement(n = 274)	*P*
Age at LT, mean (SD), y	57 (11)	58 (10)	57 (11)	0.27
Men	60.7	62.4	59.5	0.52
Race/ethnicity				0.49
White	64.1	61.6	66.1	
Black	9.5	10.5	8.8	
Hispanic	17.9	20.5	16.0	
Other	8.4	7.4	9.1	
BMI, mean (SD), kg/m^2^	29.8 (6.9)	29.9 (7.1)	29.8 (6.7)	0.81
Hypertension	55.7	59.4	52.9	0.16
BP medication use	60.0	58.9	60.6	0.783
Hyperlipidemia	26.5	27.7	25.6	0.59
Diabetes	35.5	42.6	30.3	0.006^*b*^
ASCVD	46.8	51.0	43.8	0.12
UNOS MELD at LT, mean (SD)	24.0 (10.5)	27.1 (10.3)	21.7 (10.0)	<0.0001^*b*^
LT indication				0.41
HCV	32.4	31.3	33.3	
Alcohol	22.1	22.9	21.6	
NASH	12.9	15.4	11.0	
HBV	4.7	5.0	4.4	
Autoimmune	13.1	9.4	15.8	
Cryptogenic	7.4	7.5	7.3	
Other	7.4	8.5	6.6	

Data expressed as % unless otherwise noted.

Hypertension defined by the ICD-9/10 diagnosis codes, use of BP-lowering medication, systolic blood pressure ≥140 mm Hg, or diastolic BP ≥90 mm Hg on at least 2 separate visit dates.

Hyperlipidemia defined by ICD-9/10 codes, total cholesterol ≥200 mg/dL, or use of lipid-lowering medication.

Diabetes defined by ICD-9/10 codes, A1c ≥6.5%, or use of glucose-lowering medication.

ASCVD defined by ICD-9/10 code for acute coronary syndrome, myocardial infarction, stable or unstable angina, coronary or other arterial revascularization, stroke, or transient ischemic attack.

^*a*^With or at risk for CKD was defined by ICD-9/10 diagnosis codes or estimated glomerular filtration rate <90 mL/min/1.73 m^2^ on at least 2 occasions separated by at least 90 d.

^*b*^*P* value <0.05, χ^2^ test or pooled *t* test used where appropriate.

ASCVD, atherosclerotic cardiovascular disease; BMI, body mass index; BP, blood pressure; CKD, chronic kidney disease; HBV, hepatitis B virus; HCV, hepatitis C virus; ICD-9/10, International Classification of Diseases, 9th and 10th Revisions; LT, liver transplant; MELD, model for end-stage liver disease; NASH, nonalcoholic steatohepatitis; UNOS, United Network for Organ Sharing.

### Process of Care Measures

The rates of nephrology comanagement among those with any stage or at risk for CKD decreased yearly post-LT from 35% in year 1 to 28% in year 6. Rates of nephrology comanagement among those with CKD ranged from 30% to 42% annually post-LT. Table 2 demonstrates adherence to clinical practice guidelines yearly after transplant among LTRs who were comanaged by nephrology and those who were not. Among all LTRs with or at risk for CKD, only 5%–10% had a serum creatinine and a urine albumin:creatinine ratio obtained annually as recommended by clinical practice guidelines.^10^ Less than 5% of LTRs with or at risk for CKD were offered a low-salt diet at least once yearly. Angiotensin-converting enzyme inhibitor (ACEi) or angiotensin II receptor blocker (ARB) therapy was offered to 8%–24% of LTRs with CKD and 9%–33% in those at risk for CKD. Among LTRs with or at risk for CKD and diabetes, ACEi/ARB therapy was offered to 11%–39% annually. However, in stratified analysis, LTRs who were comanaged by a nephrologist and who were at risk or had CKD and had diabetes were offered ACEi or ARB therapy at a higher frequency than those that were not comanaged by a nephrologist (12%–53% versus 9%–29% annually, respectively). There was a statistically significant difference between these annual adherence rates for 3 of the 6 post-LT y (year 2 *P* = 0.03, year 4 *P* = 0.01, year 6 *P* = 0.03). Among LTRs with or at risk for CKD and with hypertension, most had uncontrolled hypertension with only 3%–14% achieving guideline-directed average BP goal of <130/80 mm Hg annually post-LT (Table 2). Tables S1A and S1B (SDC, http://links.lww.com/TXD/A366) show yearly adherence rates to clinical practice guidelines in the subgroups of those with CKD only and the at-risk group only stratified by nephrology comanagement status.

**TABLE 2.** Adherence to clinical practice guidelines yearly after transplant among liver transplant recipients who were comanaged by nephrology compared with those who were not

		Y post liver transplant					
		1	2	3	4	5	6
Serum Cr + Ur microalbumin:Cr ratio	+ Comanagement	13/150 (8.7%)	19/141 (13.5%)	16/121 (13.2%)	7/84 (8.3%)	7/61 (11.5%)	9/38 (23.7%)
	− Comanagement	46/294 (15.6%)	28/271 (10.3%)	16/229 (7.0%)	16/198 (8.1%)	6/150 (4.0%)	4/101 (4.0%)
	*P*	0.04^*a*^	0.34	0.05	0.94	0.06	0.001^*a*^
With or at risk for CKD, offered low-salt diet	+ Comanagement	5/150 (3.3%)	5/141 (3.5%)	2/121 (1.7%)	1/84 (1.2%)	0/61(0%)	0/38(0%)
	− Comanagement	15/275 (5.5%)	11/262 (4.2%)	3/223 (1.3%)	2/196 (1.0%)	1/148 (0.7%)	2/100 (2.0%)
	*P*	0.32	0.74	1	1	1	1
With or at risk for CKD and HTN, BP <130/80 mm Hg	+ Comanagement	4/143 (2.8%)	7/132 (5.3%)	9/115 (7.8%)	9/79 (11.4%)	3/60 (5.0%)	3/38 (7.9%)
	− Comanagement	9/253 (3.6%)	16/242 (6.6%)	19/202 (9.4%)	18/177 (10.2%)	14/142 (9.9%)	14/97 (14.4%)
	*P*	0.78	0.61	0.63	0.77	0.26	0.40
At risk for CKD (eGFR, 60–89), offered ACEi/ARB	+ Comanagement	1/7 (14.3%)	1/6 (16.7%)	0/4(0 %)	1/3 (33.3%)	2/5 (40.0%)	2/3 (66.7%)
	− Comanagement	3/36 (8.3%)	2/38 (5.3%)	0/39(0%)	2/34 (5.9%)	2/22 (9.1%)	2/15 (13.3%)
	*P*	0.52	0.36	NA	0.23	0.13	0.11
CKD (eGFR, <60), offered ACEi/ARB	+ Comanagement	29/130 (22.3%)	32/129 (24.8%)	9/114 (7.9%)	23/79 (29.1%)	15/55 (27.3%)	16/35 (45.7%)
	− Comanagement	47/172 (27.3%)	39/180 (21.7%)	13/155 (8.4%)	27/141 (19.1%)	26/111 (23.4%)	20/77 (26.0%)
	*P*	0.32	0.52	0.88	0.09	0.59	0.04^*a*^
With or at risk for CKD and DM, offered ACEi/ARB	+ Comanagement	28/84 (33.3%)	29/86 (33.7%)	9/74 (12.2%)	22/51 (43.1%)	16/40 (40.0%)	16/30 (53.3%)
	− Comanagement	39/141 (27.7%)	28/135 (20.7%)	10/107 (9.3%)	19/89 (21.3%)	21/72 (29.2%)	14/48 (29.2%)
	*P*	0.37	0.03^*a*^	0.54	0.01^*a*^	0.24	0.03^*a*^

With or at risk for CKD defined as eGFR <90 mL/min/1.73 m^2^ on at least 2 occasions separated by at least 90 d.

CKD defined as eGFR <60 mL/min/1.73 m^2^ on at least 2 occasions separated by at least 90 d.

At risk for CKD defined as eGFR 60–89 mL/min/1.73 m^2^ on at least 2 occasions separated by at least 90 d.

Hypertension defined by ICD-9/10 diagnosis codes or use of BP-lowering medication or systolic BP ≥140 mm Hg or diastolic BP ≥90 mm Hg on at least 2 separate visit dates.

Diabetes defined by ICD-9/10 codes or A1c ≥6.5% or use of glucose-lowering medication.

^*a*^*P* value < 0.05. Fisher exact test or χ^2^ test used where appropriate.

ACEi, angiotensin-converting enzyme inhibitor; ARB, angiotensin II receptor blocker; BP, blood pressure; CKD, chronic kidney disease; Cr, creatinine; DM, diabetes; eGFR, estimated glomerular filtration rate; HTN, hypertension; ICD-9/10, International Classification of Diseases, 9th and 10th Revisions; Ur, urine; + comanagement, nephrology referral placed or already established with nephrologist; − comanagement, nephrology referral not placed and not already established with nephrologist.

### CV Events

During a median follow-up of 3.5 y from transplant, 14.3% of LTRs experienced a CV event. The incidence rate for CV events among LTRs with or at risk for CKD was 40.1 events per 1000 person-y of follow-up time. The unadjusted CV event incidence rate was higher among LTRs with CKD (n = 66, 47.7 events per 1000 person-y of follow-up time) compared with those at risk for CKD (n = 11, 20.5 events per 1000 person-y of follow-up time). In unadjusted analyses, comanagement by a nephrologist among LTRs with or at risk for CKD was not statistically associated with major CV events (hazard ratio, 0.67; 95% confidence interval [CI], 0.38–1.17). However, when the model was adjusted for potential confounders including age at LT, sex, race and time-varying diabetes, ASCVD, and CKD stage, nephrology comanagement was associated with a 42% lower incidence of major CV events (adjusted hazard ratio [aHR], 0.58; 95% CI, 0.42–0.82). When additionally adjusted for time-varying use of BP-lowering medications, the association was attenuated but remained significant (aHR, 0.57; 95% CI, 0.33–0.99). When the analysis was restricted to only LTRs with CKD (eGFR, <60) or only those at risk (eGFR, 60–89), nephrologist comanagement was not associated with lower major CV events (aHR, 1.05; 95% CI, 0.66–1.68 and aHR, 0.60; 95% CI, 0.27–1.34, respectively). In secondary analysis, offering of ACEi or ARB therapy among LTRs with or at risk for CKD was not associated with lower overall mortality (aHR, 1.34; 95% CI, 0.89–2.01) or major CV events (aHR, 1.31; 95% CI, 0.92–1.86). Similar findings were found among LTRs with CKD (aHR, 1.14; 95% CI, 0.69–1.90 for overall mortality and aHR, 1.03; 95% CI, 0.69–1.53 for major CV events).

## DISCUSSION

We observed a significant association between nephrology comanagement of persons with or at risk for CKD and fewer major CV events among LTRs. However, rates of nephrology referral in this population were low. In addition, rates of adherence to guideline-directed quality measures associated with CV risk reduction were almost uniformly low. These findings suggest the hypothesis that transplant provider practices need to be critically evaluated and redesigned to help facilitate comanagement of LTRs with CKD with nephrologists, which could improve adherence to CKD clinical practice guidelines and subsequent outcomes.

Current clinical practice guidelines for the general CKD population recommend nephrology referral for several indications including GFR <30 mL/min/1.73 m^2^, albuminuria, acute kidney injury, or progression of CKD.^10^ ACEi or ARB therapy is recommended for patients with CKD and evidence of proteinuria (albumin excretion, >300 mg/24 h) or diabetic nephropathy, which has been shown to improve proteinuria and delay CKD progression.^17,18^ Although studies on ACEi/ARB therapy have failed to show a reduction in major CV events when used for CKD,^17,19^ these classes of medications have been shown to improve outcomes when used for several cardiac indications including post–myocardial infarction^20^ and heart failure with reduced ejection fraction.^21,22^

Despite these guidelines, only 8.7% of patients with stage 3, 39% with stage 4, and 32% with stage 5 CKD were seeing a nephrology specialist for comanagement of CKD in the Veterans Affairs Health System.^23^ This is similar to the rates we found for nephrology comanagement of CKD among LTRs, with overall low rates of nephrology comanagement that are slightly higher for those with true CKD compared with those with CKD plus those at increased risk (30%–42% versus 28%–35%). Recent work by our group identified several barriers to providing multidisciplinary CV disease prevention care to LTRs, including (1) lack of awareness of CV disease risk after LT, (2) lack of confidence in an ability to provide proper CV disease care to LTRs, (3) reluctance to provide CVD care without transplant provider review, and (4) complexity of communication with the multidisciplinary LTRs care team about CVD care.^24^ These identified barriers provide potential targets for quality improvement initiatives to improve comanagement of prevalent CV disease risk factors, such as CKD, among LTRs.

Although comanagement of CKD and its association with clinical outcomes has not previously been studied in LTRs, it has been associated with better outcomes in the general CKD population. A systematic review of 27 longitudinal cohort studies found that early nephrology referral for management of CKD was associated with lower hospitalization and mortality rates.^9^ The results of the present study extend these findings into the LT population and show that nephrology comanagement was borderline significantly associated with a reduction in CV events, after adjusting for age, sex, race, ASCVD, and CKD stage in the CKD population. However, when the target population was expanded to include those at risk for CKD (eGFR threshold, <90), nephrology comanagement was associated with a 43% reduction in CV events independent of important confounders. If the observed association is causal, it is unclear at present how nephrology comanagement was responsible for a reduction in CV events. It could be hypothesized that LTRs comanaged by nephrology specialists might have higher rates of adherence to key clinical practice guidelines that have been demonstrated to improve clinical outcomes (eg, ACEi/ARB therapy, BP control <130/80 mm Hg). However, when further adjusting the multivariable models by use of BP-lowering medications or when stratifying adherence to clinical practice guidelines by nephrology comanagement status, we did not see any difference in rates of target BP achieved. We also did not see a significant difference in rates of ACEi/ARB therapy in those at risk for CKD compared with those with CKD. However, among LTRs with both CKD and diabetes, we did see a statistically significant difference in rates of ACEi/ARB therapy by nephrology comanagement status in 3 of the 6 posttransplant y, which could possibly explain some of the association between nephrology comanagement and CV outcomes because ACEi/ARB therapy has been shown to improve both renal and CV outcomes in the nontransplant population.^17,21^ In an attempt to further evaluate this possibility, we assessed mortality and CV outcomes by ACEi/ARB therapy, but there was not an association between offering of ACEi/ARB therapy and overall mortality or major CV events. However, the overall low rates of ACEi/ARB therapy being offered in this population limit our power to detect any associations.

Although rates of CKD in the LT population are high and increase yearly post-LT, <10% of LTRs were screened with serum creatinine and urine microalbumin:creatinine ratio yearly. Additionally, adherence rates to other key elements of the general CKD guidelines were also poor. Less than 6% of LTRs with CKD had EHR documentation that a low-salt diet was offered annually and most (88%) LTRs with CKD and hypertension did not achieve the guideline-directed average yearly BP target of <130/80 mm Hg. These findings highlight key areas for improvement in both screening for CKD and guideline-directed management of CKD among LTRs.

This study has several limitations that warrant discussion. These findings are from a single tertiary care network, and as a result, the practice patterns described here may not be generalizable to all transplant centers. However, this is the first study to report on the association between comanagement of CKD among LTRs and CV events, which are a leading cause of death after LT.^4,5,25^ Future studies should include participants from multiple institutions with diverse sex and racial-ethnic backgrounds to help make these results more generalizable to the entire LT population. Another limitation of this study is its observational nature, which does not allow us to infer causality. There is also the possibility for residual confounding and confounding by indication/referral bias that is not captured with documentation in the EHR. Another limitation is that a significant proportion of LTRs were already under the care of a nephrologist at the time of LT, and many had care encounters outside of our hospital network. Thus, there is a potential for reverse causality as date of nephrology encounters is not available in all patients. In addition, a patient was considered referred to nephrology if the patient had a referral placed in the EHR, documentation that a referral was made to nephrology, or if they were documented as currently seeing a nephrologist. A limitation of this approach is that patients seeing a nephrologist outside of our academic medical network might not have had these encounters documented, and therefore might have inappropriately been classified as not being referred to nephrology. Future prospective studies capturing actual number of encounters with nephrology specialists will be useful to identify barriers to nephrology referral for LTRs with CKD and consider possible mechanisms that may lead to reduced CV events in this population.

In conclusion, although nephrology comanagement of CKD among LTRs with CKD or at risk for CKD was associated with improved CV outcomes, rates of nephrology comanagement among this population were low. In addition, adherence rates to clinical practice guidelines for management of CKD were poor. These findings have emphasized a unique area for improvement in the clinical care of this high-risk population and a need for further study. Large, multi-institution, prospective studies would be useful to understand the generalizability of these results and identify barriers to nephrology referral and guideline-recommended management of CKD among LTRs.

## Supplementary Material


