# Supplementary material for: Comanagement With Nephrologist Care Is Associated With Fewer Cardiovascular Events Among Liver Transplant Recipients With Chronic Kidney Disease
Source: Transplant Direct. 2021 Sep 20;7(10):e766. doi: 10.1097/TXD.0000000000001220 (PMC8454906; doi:10.1097/TXD.0000000000001220)
Supplement: Supplementary file 1 [file txd-7-e766-s001.pdf]

Supplemental Table 1A

CKD ONLY (GFR <60)							
		Years Post Liver Transplant					
		1	2	3	4	5	6
Offered low salt diet	+ Co-management	5/154 (3.3%)	7/144 (4.9%)	1/124 (0.8%)	1/93 (1.1%)	0/69 (0%)	0/41 (0%)
	- Co-management	4/83 (4.8%)	0/69 (0%)	1/58 (1.7%)	1/52 (1.9%)	0/35 (0%)	0/27 (0%)
	P-value	0.55	0.06	0.58	0.67	1	1
HTN, BP <130/80mmHg	+ Co-management	4/145 (2.8%)	8/135 (5.9%)	12/115 (10.4%)	9/85 (10.6%)	3/67 (4.5%)	3/41 (7.3%)
	- Co-management	3/76 (4.0%)	5/61 (8.2%)	3/51 (5.9%)	3/45 (6.7%)	5/34 (14.7%)	4/26 (15.4%)
	P-value	0.63	0.55	0.34	0.46	0.07	0.29
DM, offered ACEi/ARB	+ Co-management	30/88 (34.1%)	22/87 (25.3%)	7/73 (9.6%)	14/52 (26.9%)	10/40 (25%)	10/27 (37%)
	- Co-management	14/35 (40%)	11/30 (36.7%)	3/23 (13.0%)	10/21 (47.6%)	8/15 (53.3%)	3/9 (33.3%)
	P-value	0.53	0.23	0.64	0.09	0.04*	0.84

Supplemental Table 1B

At Risk for CKD Only (GFR 60-90)								
		Years Post Liver Transplant						
		1	2	3	4	5	6
Offered low salt diet	+ Co-management	2/42 (4.8%)	1/46 (2.2%)	1/42 (2.5%)	0/30 (0%)	0/24 (0%)	0/16 (0%)
	- Co-management	9/146 (6.2%)	8/144 (5.6%)	2/120 (1.7%)	1/105 (1.0%)	1/81 (1.2%)	2/54 (3.7%)
	P-value	0.73	0.35	0.77	0.59	0.58	0.43
HTN, BP <130/80mmHg	+ Co-management	0/41 (0%)	1/45 (2.2%)	3/41 (7.3%)	1/30 (3.3%)	1/24 (4.2%)	2/16 (12.5%)
	- Co-management	5/133 (3.8%)	9/133 (6.8%)	9/109 (8.3%)	13/95 (13.7%)	8/77 (10.4%)	7/51 (13.7%)
	P-value	0.21	0.25	0.85	0.12	0.35	0.90
DM, offered ACEi/ARB	+ Co-management	9/27 (33.3%)	11/29 (37.9%)	4/26 (15.4%)	9/19 (47.3%)	10/17 (58.8%)	10/14 (71.4%)
	- Co-management	14/75 (18.7%)	13/75 (17.3%)	5/59 (8.4%)	8/48 (16.7%)	9/40 (22.5%)	7/28 (25%)
	P-value	0.12	0.03*	0.34	0.01*	0.01*	0.004*

*p-value < 0.05 Chi-square test or pooled t-test used where appropriate.
Abbreviations: CKD, chronic kidney disease; HTN, hypertension; BP, blood pressure; DM, diabetes mellitus; ACEi, angiotensin-converting enzyme inhibitors; ARB, angiotensin II receptor blockers.
CKD defined by eGFR < 60 mL/min/1.73m^2 on at least 2 occasions separated by at least 90 days.
At risk for CKD defined by eGFR 60-89 mL/min/1.73m^2
Hypertension defined by ICD9/10 diagnosis codes or use of blood pressure lowering medication or systolic blood pressure >= 140 or diastolic blood pressure >= 90 on at least two separate visit dates

Diabetes defined by ICD9/10 codes or A1c>=6.5% or use of glucose lowering medication
